# Type 3 Sturge-Weber Syndrome Presenting With Concurrent Epilepsy and Migraine

**DOI:** 10.7759/cureus.96262

**Published:** 2025-11-06

**Authors:** Zoreiz Z Cheema, Akawish Jahan, Nadia Siddiq, Aleena Mumtaz, Muhammad Usman

**Affiliations:** 1 Department of Medicine, Jinnah Hospital, Lahore, PAK; 2 Department of Medicine, Chaudhry Muhammad Akram Teaching and Research Hospital, Lahore, PAK; 3 Hospital Medicine, University of Wisconsin-Madison, Madison, USA

**Keywords:** focal seizures, gyriform calcifications, leptomeningeal angiomatosis, postictal hemiparesis, type 3 sturge weber syndrome

## Abstract

Sturge-Weber syndrome is a rare congenital disorder with vascular malformations affecting the brain, skin, and eyes. Type 3 Sturge-Weber syndrome, the rarest form, presents with isolated leptomeningeal angiomas, often causing diagnostic delays. This report highlights an unusual presentation where both recurrent focal seizures and migraine-like headaches were prominent, long-standing features.

A 30-year-old man presented with two focal seizures affecting the right side of his body, preceded by aura and followed by postictal confusion, right-sided weakness, and migraine-like headache with photophobia and vomiting. He had experienced focal seizures since childhood, with worsening postictal symptoms and progressive headaches over time. Examination revealed disorientation and right-sided weakness, while laboratory findings showed hyperglycemia, consistent with undiagnosed diabetes. Neuroimaging revealed leptomeningeal enhancement, tram-track calcifications, and cortical atrophy in the left parietal region, confirming type 3 Sturge-Weber syndrome. Notably, the patient had newly diagnosed diabetes, suggesting a possible metabolic link to Sturge-Weber syndrome. Management included antiepileptic therapy and glycemic control, leading to clinical improvement.

The heterogeneous neurological presentation of type 3 Sturge-Weber syndrome often delays recognition. Early neuroimaging and a high index of suspicion are essential for timely diagnosis, particularly in the absence of cutaneous features.

## Introduction

Sturge-Weber syndrome (SWS) is a rare, sporadic neurocutaneous disorder characterized by facial capillary malformation (port wine birthmark) and associated capillary-venous malformations affecting the three systems: brain, skin and eyes. The primary cause of SWS is the presence of somatic mosaic pathogenic variants in the GNAQ gene, which disrupt capillary vessel maturation during embryonic development [1]. Atypical SWS has also been linked to pathogenic variants in the GNA11 gene.

SWS is divided into three subtypes based on the presence and location of angiomas [2]. Type 1 involves both facial and leptomeningeal angiomas and may be associated with glaucoma. Type 2 is characterized by facial angiomas without intracranial involvement, though glaucoma may still occur. Type 3 presents with leptomeningeal angiomas without facial naevus and typically no ocular manifestations [3]. Type 3 SWS, though rare, presents unique diagnostic challenges due to the absence of cutaneous features, making this subtype the focus of our report.

Type 3 SWS poses a diagnostic challenge due to the absence of the characteristic facial port-wine stain, often leading to delayed or missed diagnosis. Neurological manifestations resulting from leptomeningeal angiomas may include recurrent focal seizures, progressive hemiparesis, cognitive impairment, and chronic migraine-like headaches [3]. We report a case of a type 3 SWS presenting with recurrent focal seizures, hemiparesis, and severe postictal headaches. Although both seizures and migraine-like headaches have been individually described in type 3 SWS, their persistent coexistence as prominent, long-term features is uncommon. This case underscores this unusual combination and contributes to the limited clinical literature on this rare subtype.

## Case presentation

A 30-year-old man presented to the emergency department in November 2024 with two episodes of focal seizures affecting the right half of his body. Each seizure lasted for two to three minutes, with a 20-minute gap between episodes, during which he regained full consciousness. The seizures were preceded by an aura and followed by postictal confusion and weakness on the affected side. There was no history of frothing, eye-rolling, tongue biting, or incontinence. Following the seizures, he developed a severe frontal headache, photophobia, and multiple episodes of non-projectile vomiting. He denied any fever or neck stiffness.

The patient was born at term via an uncomplicated spontaneous vaginal delivery. He had lifelong learning difficulties and memory impairment, despite normal early developmental milestones. The patient had experienced his first seizure at the age of four, though details regarding the nature of the episode were unavailable. At that time, he was treated with carbamazepine, but it was discontinued due to an allergic rash and subsequently replaced with valproic acid. Over the years, his seizures remained focal in nature, involving jerking movements of the right side of the body and transient weakness. The frequency remained relatively stable, occurring approximately once per year or once every two years. At the age of 18, his seizures became more severe, with prolonged postictal confusion lasting several days and numbness in his hands and feet. Despite these changes, they remained focal, and their frequency remained unchanged. While most episodes occurred without a clear precipitant, he occasionally identified emotional stress and low mood as triggers. The patient had a long-standing history of headaches associated with seizure episodes. These headaches had been present since childhood, typically lasting three to four days after each seizure. Over time, their severity increased, often requiring oral or intravenous medications for relief. The headaches were associated with photophobia and phonophobia but did not occur independently of seizures. There was no family history of neurocutaneous disease or epilepsy. His father was diagnosed with diabetes at the age of 50 years but had no history of hypertension or epilepsy.

On arrival at the emergency department, his vital signs were stable, with a blood pressure of 120/90 mmHg, heart rate of 88 bpm (regular), respiratory rate of 18/min, temperature of 36.7°C, and oxygen saturation of 96% on room air. However, his random blood sugar level was significantly elevated at 19.2 mmol/L (reference value: 11.1 mmol/L). On neurological examination, he was conscious but disoriented to time, place, and person, slightly drowsy, and in a postictal state, with a Glasgow Coma Scale (GCS) score of 14/15 [4]. His pupils were bilaterally equal, round, and reactive to light, and his plantar reflexes were bilaterally down going. Muscle tone and deep tendon reflexes were normal. Motor strength was 3/5 on the right side and 5/5 on the left side. Cardiovascular, abdominal, and respiratory examinations were unremarkable.

Laboratory investigations are summarized in Table 1.

**Table 1:** Results of laboratory tests TSH: Thyroid stimulating hormone.

Parameter	Patient Value	Reference Range
Complete Blood Count (CBC)		
Hemoglobin (g/dL)	15.6	13–17
Total Leukocyte Count (×10⁹/L)	12.7	4.0–11.0
Platelet Count (×10⁹/L)	163	150–450
ESR (mm/hr)	15	<20
Liver Function Tests (LFTs)		
Total Bilirubin (mg/dL)	0.4	0.2–1.2
Conjugated Bilirubin (mg/dL)	0.1	0.0–0.3
Unconjugated Bilirubin (mg/dL)	0.3	0.1–1.0
Alanine Aminotransferase – ALT (U/L)	22	7–56
Aspartate Aminotransferase – AST (U/L)	23	5–40
Alkaline Phosphatase – ALP (U/L)	98	44–147
Albumin (g/dL)	5.0	3.5–5.0
Renal Function Tests (RFTs)		
Blood Urea (mg/dL)	31	7–20
Serum Creatinine (mg/dL)	0.8	0.6–1.3
Serum Electrolytes		
Sodium (mmol/L)	138	135–145
Potassium (mmol/L)	4.0	3.5–5.0
Chloride (mmol/L)	101	98–107
Calcium (mg/dL)	10.5	8.5–10.5
Magnesium (mg/dL)	4.0	1.7–2.4
Urine Analysis		
pH	6.0	4.5–8.0
Glucose	+++	Negative
Ketones	++	Negative
Protein	+	Negative
Blood	Nil	Negative
Pus (Cells/hpf)	2–4	0–5
Leukocyte Esterase	Negative	Negative
Endocrine Profile		
TSH (µIU/mL)	2.7	0.4–4.0
Free T3 (pg/mL)	3.7	2.3–4.2
Free T4 (ng/dL)	1.4	0.8–1.8
HbA1c (%)	11.2	<5.7 (normal), 5.7–6.4 (prediabetes), ≥6.5 (diabetes)
Intact Parathyroid Hormone (pg/mL)	40.23	15–65

**Figure 1:**
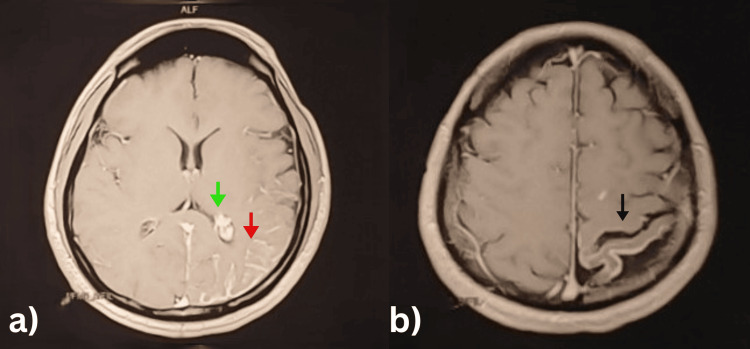
Axial post-contrast T1-weighted MRI Showing (a) enlarged choroid plexus in the lateral ventricle (green arrow) and leptomeningeal enhancement (red arrow), and (b) cortical atrophy (black arrow).

**Figure 2:**
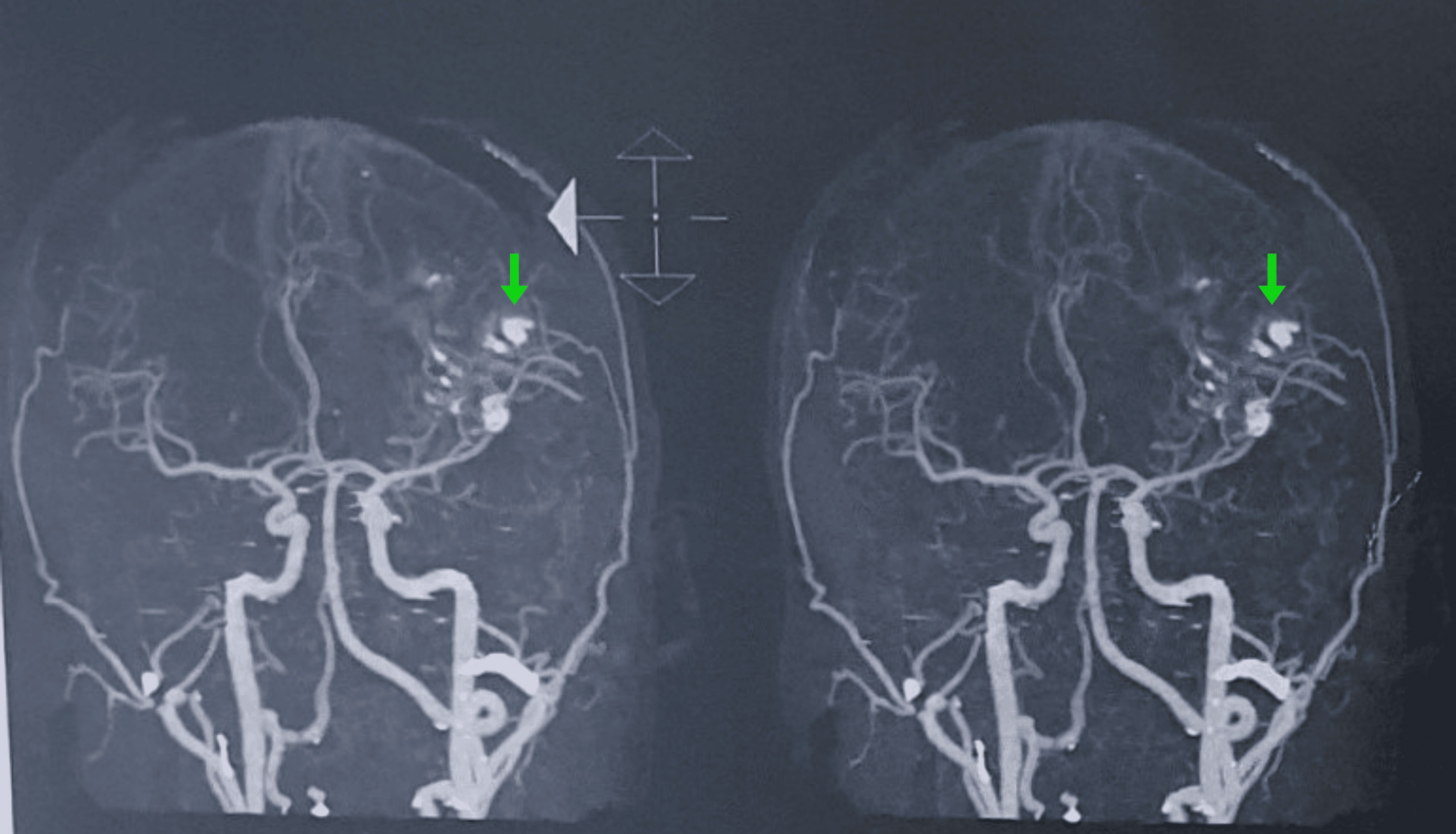
Coronal CT angiography Showing sequential sections of cerebral vessels with gyriform cortico-subcortical (“tram-track”) calcifications in the left hemisphere (green arrows).

Hematological, hepatic, renal, and thyroid parameters were within normal limits. Urinalysis showed glycosuria, ketonuria, and mild proteinuria. HbA1c was elevated, indicating poorly controlled diabetes. Viral serology was non-reactive. Neuroimaging studies revealed findings consistent with SWS (Figures 1, 2).

Non-contrast and contrast-enhanced CT brain scans showed thick gyriform (tram-track) calcifications predominantly in the left posterior parietal region, with associated cortical atrophy and mild dilation of the ipsilateral lateral ventricle. There was also contrast enhancement of leptomeningeal angiomas in the left parietal region. MRI brain with IV contrast confirmed leptomeningeal enhancement in the left parietal lobe, along with volume loss and dilated venous structures. Brain Computed Tomography Angiography (CTA) demonstrated gyriform cortico-subcortical calcifications in the left posterior parietal and occipital lobes, with significant left cerebral hemisphere atrophy.

During the hospitalization, the patient was managed with a combination of antiepileptic medications, symptomatic treatment, and stroke prophylaxis. He was administered levetiracetam 500 mg IV twice daily (BID)and valproic acid 500 mg IV three times daily (TDS) for seizure control. As the headaches were refractory to analgesics, lacosamide 50 mg orally (PO) BID was added, followed by topiramate 12.5 mg PO BID. Ondansetron 8 mg IV as needed (PRN) was given for nausea, and flunarizine 10 mg PO at bedtime (HS) was prescribed for migraine prophylaxis. To reduce stroke risk, he was started on aspirin 75 mg PO once daily (OD). At discharge, his symptoms had resolved. Levetiracetam was tapered to 500 mg PO OD due to mood changes, with plans for gradual discontinuation. Given his uncontrolled diabetes, metformin 500 mg PO BID was initiated after discharge to manage his blood glucose levels. At his one-month follow-up, the patient reported no further seizures or headaches. His blood glucose levels were well-controlled, with random blood glucose levels ranging between 8.9-10.0 mmol/L and fasting blood sugar levels between 5.6-6.7 mmol/L. Neurological examination showed a GCS score of 15/15, bilaterally equal and reactive pupils, down going plantar reflexes, and motor strength of 5/5 in all extremities [4]. He remained adherent to his seizure and diabetes medications, and his overall condition had improved.

## Discussion

Type 3 SWS is a rare variant of SWS, characterized solely by leptomeningeal angiomas without the classic facial port-wine stain or ocular manifestations. The absence of these hallmark dermatological features often leads to delayed or missed diagnosis, with patients typically first presenting with neurological symptoms. We report an adult male patient with recurrent focal seizures and migraine-like headaches, ultimately diagnosed with type 3 SWS through neuroimaging.

Seizures are the most common and earliest symptom of type 3 SWS, as seen in our patient. Prior reports confirm early-onset focal seizures, sometimes refractory to initial treatment. Tekin et al. reported an infant with refractory focal seizures and characteristic leptomeningeal angiomas, emphasizing the importance of early neuroimaging in seizure-onset cases [5]. Similarly, Aupy et al. described an adult male patient with focal inhibitory seizures and postictal paresis, highlighting the role of advanced imaging techniques like single-photon emission computerized tomography (SPECT) in detecting epileptic activity when standard electroencephalogram (EEG) is inconclusive [6]. While our patient's seizure pattern aligns with previous reports, his disease course was prolonged, characterized by persistent focal seizures over decades and progressive postictal confusion as well as cognitive decline, a presentation not extensively documented in type 3 SWS.

Apart from seizures, migraine-like headaches are another key feature of type 3 SWS. Huang et al. reported an adult female patient with intractable occipital headaches preceded by a prolonged visual aura [7]. Her imaging revealed gyriform calcifications and pial angiomatosis, confirming type 3 SWS. Similarly, Jordan et al. described a child initially diagnosed with status migrainosus, later found to have leptomeningeal angiomatosis [8]. These cases highlight the diagnostic challenge of differentiating SWS-related headaches from primary migraines, particularly in the absence of cutaneous markers. In our case, headaches were consistently postictal and worsened over time, requiring frequent analgesics. Unlike previously reported cases, our patient did not experience independent migraines, suggesting his headaches were seizure-linked rather than vascular in origin.

Visual disturbances are also common in type 3 SWS. Hayashi et al. described an adult female patient with transient right homonymous hemianopia but no history of seizures [9]. Similarly, Serindag et al. reported a young adult female patient with right-sided homonymous hemianopia following a tonic-clonic seizure [10]. In our case, the patient had no visual complaints or disturbances.

Another distinguishing feature of this case is the coexistence of type 3 SWS and diabetes, an association rarely documented in the literature. Although a direct causal relationship has not been established, hyperglycemia may affect seizure threshold and neuronal excitability, potentially influencing the disease course [11,12]. Given its potential impact on long-term disease control, further research is needed to explore the role of hyperglycemia in SWS and its implications for management.

## Conclusions

Type 3 SWS, a rare and diagnostically elusive variant, often leads to delayed recognition due to its diverse neurological manifestations and the absence of hallmark cutaneous stigmata. This case reinforces the crucial importance of maintaining a high index of clinical suspicion and pursuing early, comprehensive neuroimaging in patients with recurrent focal seizures and persistent postictal headaches, even in the absence of dermatological or ocular features. Timely diagnosis through imaging can significantly alter management strategies and long-term outcomes.

Furthermore, the coexistence of uncontrolled diabetes in this patient introduces an intriguing metabolic dimension to the disease course. The interplay between hyperglycemia, neuronal excitability, and vascular dysfunction may represent a previously underrecognized factor influencing the severity and progression of SWS. This association underscores the need for systematic exploration of metabolic comorbidities in such patients to better elucidate their role in pathophysiology and optimize multidisciplinary management.
